#  A Graphical Weighted Power Improving Multiplicity Correction Approach for SNP Selections

**DOI:** 10.2174/138920291505141106103959

**Published:** 2014-10

**Authors:** Garrett Saunders, Guifang Fu, John R Stevens

**Affiliations:** 1 Department of Mathematics and Statistics, Utah State University, Logan, UT 84322, USA

**Keywords:** Graphical weighted approach, Multiple correction, Hypothesis testing, QTL, Linkage disequilibrium.

## Abstract

Controlling for the multiplicity effect is an essential part of determining statistical significance in large-scale single-locus association genome scans on Single Nucleotide Polymorphisms (SNPs). Bonferroni adjustment is a commonly used approach due to its simplicity, but is conservative and has low power for large-scale tests. The permutation test, which is a powerful and popular tool, is computationally expensive and may mislead in the presence of family structure. We propose a computationally efficient and powerful multiple testing correction approach for Linkage Disequilibrium (LD) based Quantitative Trait Loci (QTL) mapping on the basis of graphical weighted-Bonferroni methods. The proposed multiplicity adjustment method synthesizes weighted Bonferroni-based closed testing procedures into a powerful and versatile graphical approach. By tailoring different priorities for the two hypothesis tests involved in LD based QTL mapping, we are able to increase power and maintain computational efficiency and conceptual simplicity. The proposed approach enables strong control of the familywise error rate (FWER). The performance of the proposed approach as compared to the standard Bonferroni correction is illustrated by simulation and real data. We observe a consistent and moderate increase in power under all simulated circumstances, among different sample sizes, heritabilities, and number of SNPs. We also applied the proposed method to a real outbred mouse HDL cholesterol QTL mapping project where we detected the significant QTLs that were highlighted in the literature, while still ensuring strong control of the FWER.

## INTRODUCTION

Linkage Disequilibrium (LD) analysis plays a fundamental role in gene mapping, as a tool for uncovering biological trait regulating genes. Many biological traits are influenced by genetic variants and hence it is possible to determine the rough genomic position of the causative variations through associations between SNPs and phenotype [1-13]. Among the popular SNP selection approaches, the single-locus association with solid multiplicity correction ability remains a powerful tool as associations can generally only be found over small distances [14]. Moreover, as tens-of-thousands of SNPs for genome-wide association studies (GWAS) are under demand [5], the single SNP based LD analysis can at least provide a necessary initial screening selection to detect a subset of promising candidates for further exploration [15, 16].

Despite the great progress which has already been made within LD based Quantitative Trait Loci (QTL) mapping, powerful and computationally efficient methods for large-scale simultaneous testing of individual SNPs with strong control of the familywise error rate (FWER) are still lacking [17, 18]. FWER is the most accepted error rate used to determine significance levels for large-scale testing problemswhere the goal is to provide conclusive results. In some studies, researchers often control the False Discovery Rate (FDR) to obtain a large pool of potentially significant SNPs and then select only the most significant subset for validation due to cost restrictions. However , this rule can lead to unwanted results as the FDR is controlled only for *all* selected SNPs , and provides no promise of control for an arbitrarily selected subset of the significant SNPs. Thus, we recommend controlling the FWER (in place of the FDR) in exploratory scenarios where only the most promising results will be considered.

The Bonferroni correction, as one of the most widely used statistical procedures, is often employed to control the FWER when multiple tests are conducted. However, the Bonferroni correction is not favorable in large-scale testing because it substantially reduces the statistical power, hence decreasing the chances of detecting SNPs with real effects [19]. While permutation procedure has been widely employed to adjust for correlated tests, it is computationally expensive [15, 20, 21] and may mislead in the presence of family structure [22]. Moreover, the permutation approach was designed for only one test setting. In LD based QTL studies, the high likelihood of dependencies among SNPs and the two tests structure strongly demand a new multiplicity adjustment approach that maintains simplicity but is more powerful.

In the LD based QTL model [13], detecting a significant QTL that is associated with a certain phenotype requires two hypothesis tests, one testing for the existence of a QTL for a given SNP (i.e. whether or not the QTL is associated with the phenotype), and the other testing for the strength of the LD between the SNP and the existing QTL (i.e. whether or not the QTL is successfully detected by the model). Although the existence of a significant QTL is the ultimate goal, the degree of LD between the QTL and SNP is also critical in guaranteeing the basic assumptions of the model. By Fu *et al.*'s assumptions [13], the unobservable QTL can be mapped by its association with the observable SNP through the conditional probability of the genotype of the QTL given the genotype of the SNP. Therefore, only QTLs that are not only significantly existing but also strongly linked with a SNP will be identified, i.e. identifying a significant QTL requires rejecting these two hypotheses simultaneously. Although the LD based QTL model has been successful in locating significant QTLs [13, 23, 24], the Bonferroni multiplicity correction approach used previously ignored two important issues. First, if the QTL existence test fails to reject, then the LD between the SNP and QTL is not identifiable in their mixture model. Second, the Bonferroni correction is too conservative for large-scale of SNPs.

In this article, we propose a new power improving multiplicity correction approach specially designed for the LD based QTL mapping, on the basis of graphical weighted-Bonferroni methods [25]. By introducing a logical structuring for the two tests involved for each SNP, i.e. higher order for QTL existence testing (primary) than the LD testing (secondary), the LD test will never be investigated if the primary test concludes that the QTL does not exist. By exploiting the priority ordering of the two hypotheses to adjust the p-values, our proposed approach can avoid the previously mentioned identifiability issue, and address the multiplicity correction for large-scale number of SNPs. None of the current LD based QTL methodologies directly overcome these challenges when performing these two tests [13, 23, 24]. Our proposed multiple correction approach with priority structuring has been shown to synthesize weighted Bonferroni-based closed testing procedures such as the weighted Bonferroni-Holm procedure, fixed sequence tests, gatekeeping procedures, and the fallback procedure into a powerful and versatile graphical approach [25], which we tailor here for the LD based QTL mapping.

In the following section we present the LD based QTL model and the two tests involved. Next, we describe in detail how we design the logical structuring to perform the multiplicity correction for the LD based QTL model. The significance of the power advantage of the proposed method over the Bonferroni correction is established through both simulations and one real QTL mapping project. Since sample size, heritability, and number of SNPs all determine the power of the method, we illustrate the power through heritability of 0.1 and 0.4, sample size small (100), medium (300), and large (500), and number of SNPs changing from 1, 10, 50, 100, 500, to 1,000. We end with a discussion of the results.

## METHODOLOGY

## LD Based QTL Mapping Model

To map the rough location of the QTL regulating a certain biological trait, we apply the mixture model [13]. Under this model, QTL is detected by statistically modeling the genotypic variation through not only the association between phenotype and the putative QTL, but also the association between the putative QTL and SNP. Since the SNP genotype is observable, we can infer the probabilities of a putative QTL genotype by the conditional probability of QTL (* A*) genotype given the SNP (* M*) genotype, as long as there exists LD between the SNP and putative QTL [26].

The mixture model of [13] assumes each individual's phenotype 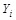 , 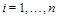 , is a random variable from density 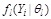 , where 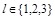 denotes three distinct QTL genotypes. Each QTL genotype is assumed to induce a separate distribution of phenotypes. Typically, normal distributions are assumed for each 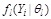 with 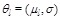 . From these assumptions, the corresponding likelihood is expressed as [13]


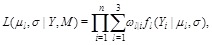
 (**1**)

where 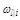 is the conditional probability of individual 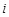 having QTL genotype 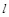 given their SNP genotypes , 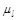 is the phenotypic mean for QTL genotype 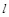 , 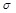 is the common standard deviation for all genotypes, and 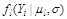 is the probability density of observations for individual 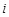 at QTL genotype 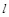 [13, 26, 28].

The probability of the SNP's major allele (* M*) is denoted by 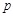 , and correspondingly 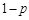 for the minor allele (* m*). Similarly, the probability of the QTL's major allele (* A*) is denoted by 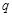 , and correspondingly 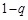 for the minor allele (* a*). Together, the SNP and QTL form four haplotypes (* MA, Ma, mA*, and *ma*) with corresponding frequencies 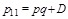 , 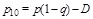 , 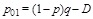 , and 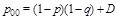 , respectively. Here, 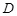 is the linkage disequilibrium between SNP and QTL. The conditional probabilities 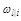 of the QTL's various genotypes (* AA, Aa*, and *aa*) can be calculated upon the observed SNP genotypes (* MM, Mm*, and *mm*) from the joint probabilities [13, 27]. Hence, 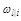 is a function of 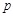 , 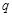 , and 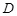 . The EM algorithm is then applied to the likelihood in (1) to obtain maximum likelihood estimates for all parameters [13, 27].

## Two Hypothesis Tests

Through the likelihood in (1), the hypotheses 


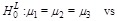



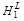
 : one of the equalities above does not hold (**2**)

can be used to test if the QTL is significantly associated with phenotype 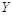 ( i.e. existence of QTL ). Since all the unknown parameters in (1) were estimated by maximum likelihood estimates (MLEs), a log likelihood ratio statistic can be used to test the hypotheses in (2) [13]. The resulting test statistic ( 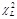 ) is asymptotically distributed as a 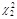 under 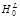 for large enough samples [13, 26].

On the other hand, linkage disequilibrium, denoted by 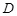 , between the SNP and QTL can be tested by means of the hypotheses


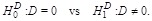
 (**3**)

Once the existence of a QTL is established, the test statistic used to judge whether or not the QTL is significantly associated with SNP is [13, 29]:


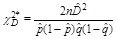
 (**4**)


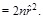
 (**5**)

Here, 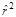 is the square of the correlation coefficient between the SNP and QTL that has been used in most of the related literature, which has many good sampling properties [30, 31]. Under 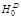 , 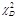 is asymptotically distributed as 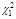 , from which the tail probability (p-value) of the observed level of association can be determined [10, 23, 26, 29, 32].

Because whether or not a QTL exists and whether or not the existing QTL is able to be successfully identified by the model are both critical, the QTL will not be interpreted as significant unless the two hypothesis tests (2) and (3) are both rejected. Three problems urge the improvement on this two hypothesis tests setting. Firstly, 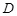 is not identifiable under the null hypothesis 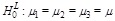 [33, 34]. That is, the parameter 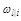 falls out of the model when the means are equal, as the 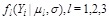 in the likelihood (1) are identical in this case, resulting in 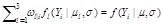 . Hence, the likelihood reduces to 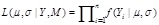 , so that 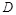 which is computed from 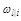 can not be computed. As a result, testing 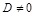 under 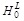 is not meaningful. Secondly, a multiplicity correction is needed for simultaneous testing of all SNPs. Thirdly, the existence of a QTL underlying certain biological traits is the ultimate goal for the real application. Inspired by the idea of the graphical Bonferroni approach [25], we set the existence of QTL (2) to be the primary test and the LD test (3) to be the secondary test. If the primary test is not rejected, the secondary test will not be investigated. As a result, our proposed multiplicity correction approach increases the power, while preserving strong control of FWER and avoiding the identifiability issue of 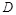 under 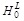 .

## Graphical Bonferroni Approach

The Graphical Bonferroni Approach (GBA) is a versatile and easily communicated general adjustment method for multiple testing [25]. Provided as a generalized framework, it must be specially tailored to each testing situation. Generally speaking, it is most powerful for situations where hypotheses can be partitioned into levels of importance such that the most important hypotheses are tested first and the lower level hypotheses are tested only if the higher level hypotheses show significant results.

All hypotheses of interest are depicted as nodes in a directed acyclic graph. Local significance thresholds for each node (hypothesis) dictate the local level at which each hypothesis is tested. Weighted edges between all nodes map the logical structuring of the designated testing approach. When a hypothesis is rejected, the weighted edges dictate the proportion of the locally assigned significance threshold that is passed from the rejected node to all connected nodes. Thus, the graph induces an iterative testing approach that is shown to result in a closed-test that admits a short-cut [25]. Further, Algorithm 1 of [25] provides a simple updating technique that performs the short-cut. Strong control of the FWER at level 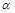 is proven to occur so long as three regularity conditions are met: 1) the sum of the local significance thresholds is no more than 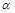 , 2) the sum of outgoing edge weights from each node are no larger than unity, and 3) no node has an edge connecting to itself [25].

## Rejection Scheme

**Fig. (1):**
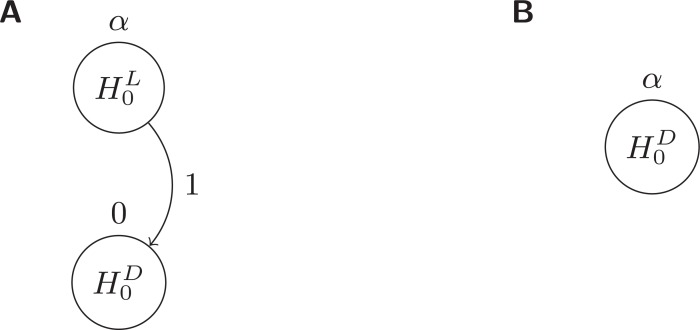
1

Since the ultimate goal is to check existence of QTL, the first interest is in testing 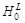 in (2) to see if the phenotype shows evidence of association with a latent QTL. Depending on the results of the test of (2), the testing for the given SNP will either end, or interest will be turned to testing 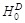 in (3) to see if the SNP is also associated with the QTL. (Fig. **1A**) demonstrates how all of 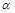 is used to test the first hypothesis, 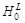 , and none of 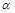 is initially given to the testing of 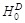 . That is, node 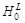 has local significance threshold 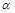 , and 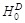 has local significance threshold 0. Assuming 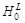 is claimed significant, the node belonging to 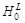 would be removed and all of 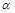 passed on to 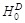 as signified by the edge weight of 1 along the path from 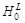 to 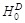 . At this point, 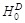 is tested at level 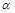 , its new local significance threshold given the rejection of 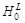 as shown in (Fig. **1B**).

Note that adjusted 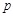 -values could be similarly obtained for each node. The adjusted 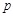 -value for 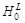 would be the same as the unadjusted value. The adjusted 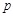 -value for 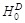 would be either larger of its unadjusted value and 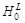 's unadjusted value (if 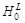 was significant at level 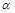 ) or 1 (if 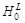 was not significant at level 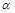 ). The structuring ensures that a child node (such as 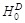 in Fig. **1**) cannot have a smaller adjusted 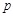 -value than its parent node ( 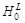 in Fig. **1**).

As a result, for the single SNP analysis, either both hypotheses will be tested at level 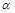 , or the testing will stop after 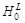 without considering 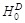 . Alternatively, the Bonferroni correction would test both hypotheses at 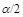 . Hence, the Bonferroni adjustment has less power, due to its smaller thresholds. Compared to the traditional Bonferroni, the only potential disadvantage of the GBA method is that it skips testing 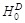 if 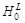 is not significant. However, this potential disadvantage becomes an advantage for the LD based QTL model because 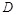 is not identifiable under the null of 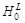 . Thus, the only situation in which the Bonferroni method would have a possible advantage over the GBA method is not applicable here.

Our proposed GBA method further achieves an advantage in the case of multiple SNPs through sharing of the 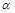 -level between SNPs. Say there are 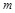 SNPs to be tested for both 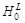 and 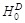 . Let 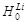 and 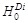 denote these two hypotheses respectively for the 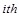 SNP, 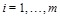 . The GBA approach begins by allocating initial local thresholds of 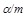 to each 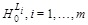 . Initial local thresholds of zero are allocated to each 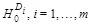 . Edge weights of 1 are established from each 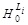 to the corresponding 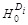 for all 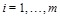 . Edges with weight 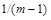 are also established between each 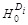 and all 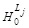 for each 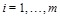 and 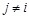 . Multiple testing (or multiplicity adjustment) then proceeds according to Algorithm 1 of [25].

**Fig. (2):**
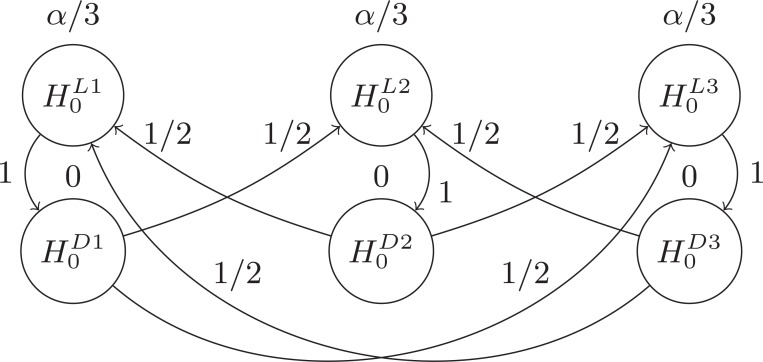
2

Fig. **2** demonstrates the case of multiple SNPs, taking 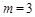 as an example for simplicity. In addition to the schemes demonstrated in Fig. **1**, Fig. **2** shows two more rules. First, it includes the extra edge weights from each 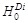 node to all non-parent 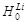 nodes, i.e. to all 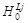 with 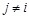 . This allows for additional 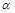 -sharing between SNPs when both hypotheses (i.e. 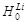 and 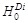 ) are rejected for any given SNP 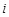 . Second, the 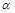 -level is split with a Bonferroni type allocation between the 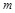 top-level hypotheses while none of 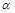 is initially provided to the 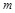 lower-level hypotheses. Upon rejection of a higher-level hypothesis, the lower-level child hypothesis receives all of the 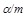 -level of the parent (edge weight of 1). If the lower-level child hypothesis is then also found to be significant, its 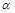 threshold is then shared between all remaining higher-level hypotheses (edge weights of 1/2).

**Fig. (3):**
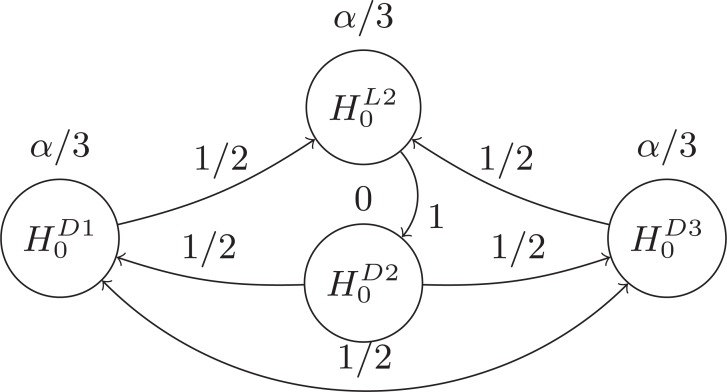
3

The power advantage of our proposed GBA over the Bonferroni method is evident from the larger thresholds. Where the Bonferroni method would test each hypothesis at the 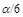 -level, the GBA tests each hypothesis by thresholds that are no smaller than 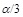 . To demonstrate, assume that 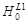 and 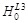 from Fig. **2** are rejected at the 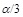 level, but that 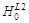 is not. Then nodes corresponding to the rejected hypotheses 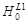 and 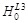 are removed and all 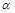 thresholds and edge weights are updated as shown in Fig. **3**. Notice in Fig. **3** the reconnecting of edge weights which previously attached to 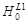 and 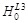 from 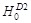 , 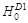 , and 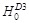 . This demonstrates how edges determine not only the weight that will be passed, but also define the inheritance of edge weights.

**Fig. (4):**
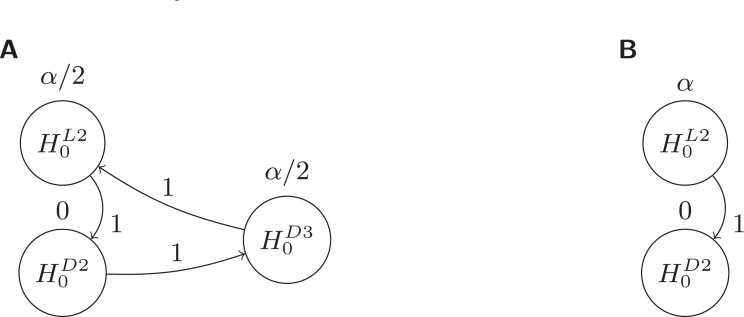
4

Assume now that 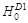 of Fig. **3** can be rejected at the 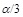 -level. The graph updating (Fig. **4A**) becomes more complicated with this rejection because the rejected hypothesis is both sending out and taking in edge weight from the same hypotheses (nodes). Specifically, 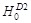 is set to send half of its threshold to 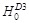 and the other half to 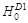 . Of the half that the now rejected 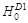 would have received from 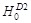 , half is designated to 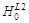 and the other half designated to go to 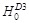 . This assignment causes the updated 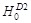 to send a total weight of 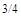 to 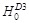 . However, recalling the logical structure of the hypotheses, it can be seen that 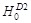 will not be considered for testing unless 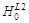 is first rejected. Hence, the 1/4 that 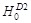 would pass on to 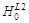 through 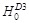 at this point is not logically possible as this would require testing 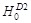 before testing 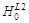 . This logical restriction allows us to move the 1/4 out from 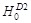 by means of the only other path available, so that 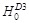 receives a total weight of 1 from 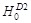 , as shown in Fig. **4A**.

The node corresponding to 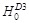 in Fig. **3** was sending half of its threshold to 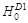 and the other half to 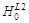 . With the removal of 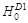 , now assumed to be significant, 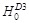 will now be doubly joined to 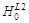 and to itself by inheriting the outgoing paths from 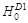 to both 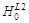 and 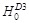 . This junction of 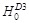 to itself would specifiy that the 1/2 that was going from 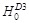 to 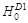 times the 1/2 that was going from 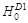 to 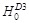 would result in 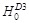 returning 1/4 to itself. Since it is not possible for 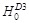 to pass 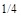 back to itself, it passes to 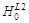 the original 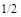 it was already sending to 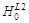 , plus the 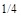 inherited by 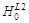 from 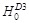 via 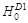 plus the 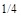 that 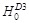 re-inherited from 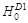 . The result is to have 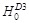 send all of its threshold to 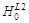 . This can also be viewed more simply by the fact that upon removal of 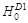 from the graph (due to its rejection), 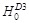 is left with only one outgoing edge to 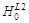 , hence all of its threshold must be passed to 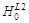 .

The final graph resulting from the rejection of 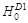 in Fig. **3** is depicted in Fig. **4A**. At this point it could be possible that 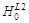 is rejected, but to demonstrate a more interesting scenario, assume that 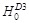 only can be rejected at the 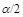 -level. The resulting graph with 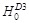 removed is depicted in Fig. **4B**. Interestingly, both 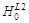 and (if significant) 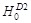 can now be tested at the full level 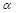 .

In conclusion, we solve the three problems in the multiplicity testing scenario that exist in previous LD based QTL models. Firstly, the unidentifiable issue of 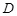 under the null hypothesis 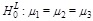 is avoided by skipping testing 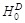 when 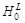 is not rejected. Secondly, by flexibly passing out different portions of significance level 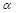 according to the conclusion of other tests, we make a correction for all multiple SNPs. Thirdly, by setting 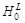 to be the primary test over 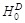 , we satisfy the real application concern that existence of QTL is the ultimate goal. In the remainder of this article, we show through simulation studies that the proposed GBA is more powerful for LD based QTL mapping than standard Bonferroni adjustments and thus leads to more scientific discovery while maintaining strong control of the FWER.

## RESULTS

## Power Simulation

We investigated a simulation study to quantify the power advantage of the proposed graphical Bonferroni approach (GBA) over the standard Bonferroni adjustment within the LD based QTL mapping model [13]. The QTL, phenotype, and SNPs were generated under the assumptions of the alternative hypotheses (described in Methodology section in (2) and (3) ). The QTL was generated using an assigned probability of 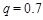 for the major allele. For each individual 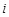 , 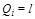 with 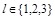 was used to code the QTL genotypes of *aa, Aa*, and *AA*, respectively. The normally distributed phenotype dependent on the value of the QTL is generated as 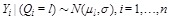 . The means for the phenotype 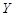 corresponding to the values of the QTL were set at 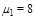 , 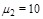 and 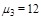 . SNPs were then generated using the conditional probability of the SNP genotype given the value of the QTL genotype for each individual. In general, for an LD based QTL mapping model, researchers genotype the SNP first and then use the SNP to generate a QTL based on the conditional probability of QTL genotype given SNP genotype. However, for our purposes, we are interested in extending from single SNP mapping to multiple SNPs mapping. Therefore, we derive the conditional probability of SNP genotype given QTL genotype (see Table **1**) from the Bayes Rule in Equation (6).


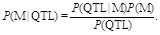
 (**6**)

Sample sizes of 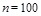 , 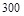 , and 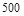 were used to represent small, medium, and large sample sizes, respectively. The number of SNPs per simulation was set at 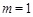 , 10, 50, 100, 500, and 1,000 to show the initial power under the single SNP scenario and the corresponding decreasing power trend as the number of SNPs increases. Finally, the heritability was set at two values, 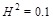 and 0.4, corresponding to high and low error variance [27]. The model error variance 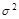 was computed using the heritability and genetic variance of the QTL. Power estimates were averaged over 1,000 simulations.

**Fig. (5):**
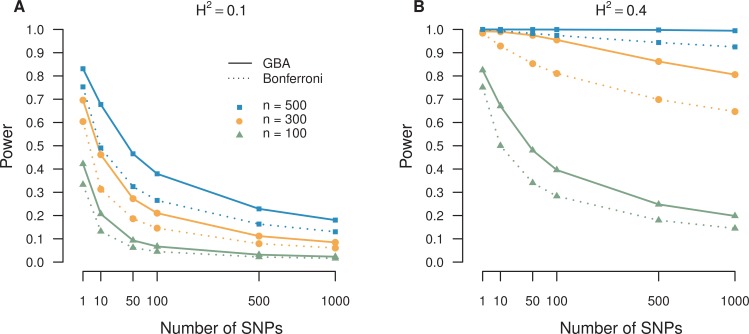
5

The simulation results, shown in Table **2** and depicted in Fig. **5**, demonstrate the power comparison of the proposed GBA with the traditional Bonferroni adjustment. The traditional Bonferroni adjustment approach makes corrections not only for multiple SNPs but also for two tests of each SNP, with total 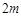 number of tests. Here 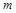 is number of SNPs. Our proposed GBA performs number of corrections somewhere between 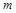 and 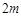 , flexibly depending on the real situation of each SNP. The designed simulation results also provides an experimental reference for researchers about how power varies among different sample size 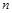 , the number of SNPs 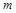 , and the degree of heritability 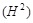 . As expected, the power under high heritability (B: 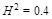 ) is much higher than that of the low heritability (A: 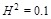 ) and the power under large sample size ( 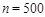 , blue curves ) is much higher than that of the small sample size ( 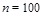 , green curves ). Under high heritability ( 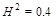 ) and a larger sample size ( 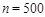 ) , the power of the multiplicity adjustment remains high even as the number of SNPs becomes large ( 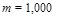 ). However, in practice it is often expensive to collect so many sample measurements. It is worth mentioning that the power obtained from the GBA can achieve 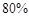 for a large number of SNPs ( 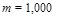 ) but medium sample size ( 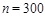 ). Moreover, even with a low heritability ( 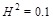 ), the power increase of the GBA over the Bonferroni adjustment allows for the possibility of maintaining the power level of the Bonferroni adjustment while decreasing the sample size of the study or increasing the number of SNPs, a great advantage for researchers. For example, under many cases, the power of our improved approach for 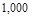 SNPs is similar or larger than the power of the Bonferroni adjustment for 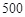 SNPs.

Although the power increase of our proposed method improves moderately over the standard Bonferroni adjustment for the case of low heritability ( 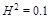 ) when the sample size is small ( 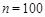 ), the power gains are still comparable to seminal results found by previous multiplicity improvements over their competitors [35]. All in all, our proposed GBA method shows a substantial increase in power over the Bonferroni adjustment under all 12 circumstances with the different combinations of sample size, number of SNPs, and heritability. We did not compare with permutation because it is computationally infeasible to compute these six settings using permutation. In addition, permutation approach is designed for the one testing structure, which is not the case here. Finally, we firmly believe that the GBA works better in the LD based QTL model because of the priority order setting, the logical consistency, uniformly better power in theory, and increase in computational efficiency.

## Mouse HDL Cholesterol QTL Mapping Project

Epidemiological studies have consistently shown that the level of plasma high density lipoprotein (HDL) cholesterol is negatively correlated with the risks of coronary artery disease and gallstones [36, 41]. Because of the inverse relationship between HDL and cardiovascular disease, there has been considerable interest in understanding genetic mechanisms contributing to variations in HDL levels. HDL levels vary considerably in different people, which are affected by interactions of multiple genes and environmental factors, and up to 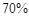 of this variation in humans is genetically determined [37, 42]. Because of the concordance between human QTLs regulating HDL and corresponding mouse loci and many easily controlled experimental advantages, mouse has become an animal model in HDL research. Numerous findings in HDL QTL associations are obtained from crosses between different inbred mouse strains. By crossing inbred strains that significantly differ in HDL levels and subsequently testing for association between HDL levels and genetic SNPs in the progeny, numerous significant QTLs involved in HDL have been identified in mouse [36, 41, 43, 47].

Compared to the inbred mice strains with coarse mapping resolution, the QTL research on wild-caught and commercial stocks of outbred mice, as resources for genetic fine mapping, is far under developed. Zhang *et al.* published an open resource outbred mouse database with 288 Naval Medical Research Institute (NMRI) mice and 44,428 unique SNP genotypes (available at http://cgd.jax.org/datasets/ datasets.shtml) [48]. Three hundred 4-to-6-week-old male NMRI mice were purchased and individually housed with the same diet and environmental conditions. The blood samples of each mouse were measured by submandibular puncture after a 4-hr fast. Then plasma samples were frozen for measurement of HDL cholesterol. There were 10 mice removed because the standard deviation of individual blood pressure is greater than two. Another two mice were also discarded for their 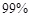 identity of SNP genotypes. This caused the final sample size to be 288. A total of 581,672 high density SNP were initially genotyped by the Novartis Genomics Factory using the Mouse Diversity Genotyping Array [49]. In order to guarantee promising data for association mapping studies [50], only polymorphic SNPs with minor allele frequency greater than 2%, Hardy-Weinberg equilibrium 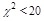 , and missing values less than 40% were retained. Moreover, identical SNPs within a 2Mb interval were collapsed. This left 44,428 unique SNP genotypes for their resulting analysis using three analysis methods, linear trend test, two way ANOVA, and EMMA [51]. From Zhang's work , adjustments for multiplicity at the genome-wide association level were made using a simulation approach [52] as well as the permutation approach [53]. They identified three loci as significant, with two loci on Chromosome1 (Chr1) and a single locus on Chromosome5 (Chr5) (see Fig. **3** of [48]). However, after a closer investigation, Zhang *et al.* reported that the significant findings in Mb182 of Chr1 is spurious.

**Fig. (6):**
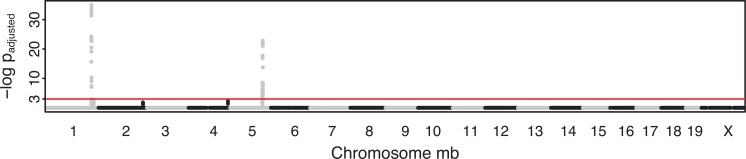
6

We applied the introduced LD based QTL model [13] and the proposed GBA multiplicity correction approach to this outbred mouse HDL cholesterol genome data to compare our findings with the highly validated discoveries in current literature. Recalling the detailed adjustment structure of the GBA, it can be seen that the adjusted 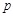 -value obtained from GBA for the test of 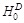 will never be smaller than that of 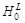 . Hence, reporting the significant adjusted 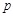 -values for 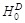 is sufficient for demonstrating those SNPs that show strong evidence of linkage to a significantly existed QTL. (Fig. **6**) depicts the negative log of the adjusted 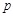 -values for 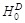 for each SNP as a function of the location (in Mb) of each SNP for 19 chromosomes. The threshold for the adjusted 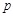 -values of 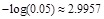 supports two dramatically significant findings, on Chr1 at Mb173 and Chr5 at Mb125. These two significant discoveries are the same as the findings in current outbred mouse literature, compare to Fig. **3** of [48], but with an even stronger signal.

In Table **3** we notice that all significant QTLs detected from outbred mouse by our model are confirmed from reported findings obtained from inbred mouse crosses using very different approaches. Two QTLs have been reported coincident with candidate genes. Chr1 locus at Mb173, the highest peak in Fig. **6**, is the major determinant of HDL, which has been detected as QTL *Hdlq15* in inbred mouse strains multiple times (as referenced in Table **3**). Combining mouse crosses with haplotype analysis for the HDL QTL located on Chr 1 locus at Mb173 reduced the list of candidates to a small amount. Numerous mouse crosses have linked HDL to this region, and *Apoa2* has been identified as the gene underlying the QTL [38, 40, 41, 43, 46]; this gene has been highlighted in *Nature Reviews Genetics* [54]. Chr5 locus at Mb125, the second highest peak in Fig. **6**, is located in the same locus as QTL *Hdlq1* found by [46] and [44] (as referenced in Table **3**). In addition, they conclude that *Scarb1* (a well known gene involved in HDL metabolism) is the causal gene underlying *Hdlq1* by haplotype analysis, gene sequencing, expression studies, and a spontaneous mutation [45, 47].

There are a total of two significant detections from a large pool of 44,428 candidate SNPs from our model, with the findings confirmed by inbred mouse findings. Zhang *et al.* worked on exactly the same data, adjusting for multiplicity at the genome-wide association level using a simulation approach [52] as well as the permutation approach [53]. They made the same two significant detections with less significance level than our p-values, and they also find one spurious QTL. Therefore, our proposed approach brings a useful alternative approach for SNP selection literature.

## DISCUSSION

Detecting significant genes that cause disease (for example the inverse relation between human cholesterol and cardiovascular disease) or regulate biological traits through LD based QTL mapping has been popular in many disciplines [1-13]. The new techniques can simultaneously consider tens of thousands of SNPs and hence bring big challenges to multiple testing. In addition, high dimensional biological traits, often reduced to multiple PC components, have been widely used and add yet another demand for a powerful and computationally efficient approach to adjust for multiple tests [13, 55-57].

These multiple tests require an adjustment on the resulting 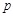 -values in order to preserve control of the familywise error rate (FWER) at a pre-specified level 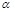 . In some cases, follow up work on the significant findings may justify using the false discovery rate (FDR) as the error rate of interest. Typically however, the significant results are directly reported and therefore the FWER is the more desirable form of error rate to control. The current standard approach in LD based QTL mapping is to apply a Bonferroni adjustment to correct for multiplicity and preserve the FWER. As is well known, the Bonferroni correction is overly conservative for large numbers of tests, but the advantages of simplicity without independence assumptions on the corresponding family of tests continue to make it popular.

In this article, we tailored a multiple correction approach, based on graphical weighted-Bonferroni methods [25], which allows for the logical order among the two hypotheses to be structured into the multiplicity correction. As in the LD based QTL mapping model of [13], we need to test two hypotheses for each SNP, one with 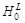 about whether or not an association exists between QTL and phenotype, and the other with 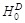 about whether or not LD exists between SNP and QTL. Among these two tests, the QTL existence test has higher priority because the LD test will not be applicable if a QTL does not exist, and the existence of QTL is the ultimate goal in real applications. Although the logical structure of the two tests is known , none of the current LD based QTL literature considers this priority structure when performing these two tests [13, 23, 24, 27]. By the structure of graphical weighted Bonferroni, the quantitative trait loci test and linkage disequilibrium test are integrated into a combined multiple testing correction framework [58]. As a result, GBA approach has more potential applications in QTL studies. For example, in a haplotype study, we can put QTL test, dominant or additive effect test into one multiple testing correction framework. Hence, if QTL test is not significant, we don't have to test dominant or additive effects.

The significance of the power advantage of the proposed method, established through simulations, and finally on real data, is such that we advocate its use whenever multiple tests are needed for the LD based QTL mapping design, where both 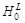 and 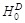 tests are considered.

## Figures and Tables

**Table 1.** The theoretical conditional probabilities of SNP genotype (columns) given QTL genotype (rows).

	*MM*	*Mm*	*mm*
*AA*	$\frac{p_{11}^{2}}{q^{2}}$	$\frac{2p_{11}p_{01}}{q^{2}}$	$\frac{p_{01}^{2}}{q^{2}}$
*Aa*	$\frac{2p_{11}p_{10}}{q\left(1-q\right)}$	$\frac{2\left(p_{11}p_{00}+p_{10}p_{01}\right)}{q\left(1-q\right)}$	$\frac{2p_{10}p_{00}}{q\left(1-q\right)}$
*aa*	$\frac{p_{10}^{2}}{{\left(1-q\right)}^{2}}$	$\frac{2p_{10}p_{00}}{{\left(1-q\right)}^{2}}$	$\frac{p_{00}^{2}}{{\left(1-q\right)}^{2}}$

**Table 2.** The results of the power simulation as depicted in Figure 5.

	n = 100		n = 300		n = 500	
m	Bon.	GBA	Bon.	GBA	Bon.	GBA
H^2^ = 0.1						
1	0.333	0.422	0.604	0.696	0.753	0.831
10	0.132	0.207	0.313	0.462	0.490	0.677
50	0.062	0.093	0.186	0.272	0.324	0.465
100	0.045	0.067	0.146	0.210	0.265	0.379
500	0.022	0.032	0.079	0.112	0.163	0.229
1000	0.016	0.023	0.060	0.085	0.130	0.180
H^2^ = 0.4						
1	0.751	0.825	0.984	0.992	1.000	1.000
10	0.500	0.671	0.929	0.990	0.994	1.000
50	0.340	0.480	0.853	0.975	0.983	1.000
100	0.283	0.396	0.811	0.955	0.974	0.999
500	0.180	0.248	0.699	0.862	0.944	0.998
1000	0.145	0.198	0.647	0.806	0.925	0.994

**Table 3.** The significant results of the outbred mice HDL cholesterol QTL mapping project depicted in Figure 6. SNPs are ordered by significance level. Corresponding concurrence candidate gene and QTL from previous inbred crosses studies are shown.

Chr	Position (Mb) 5pt	Adjusted P 5pt	Raw P 5pt	Raw P	Candidate	Inbred	Ref.
		(H^D^_0_)	(H^L^_0_)	(H^D^_0_)	Gene	QTL	
1***	173,155,512	5.7 x 10 - 15	1.3 x 10-19	3.0 x 10-30	*Apoa2*	*Hdlq15*	[38,41,46,47,54]
5***	125,530,593	5.2 x 10 - 10	1.2 x 10 - 14	2.0 x 10 - 83	*Scarb1*	*Hdlq1*	[37,40,44-47]

*** Significant at the FWER 5*10-10 level.
